# Supplementary material for: Metabolomics-Based Analysis of the Effects of Different Cultivation Strategies on Metabolites of Dendrobium officinale Kimura et Migo
Source: Metabolites. 2023 Mar 6;13(3):389. doi: 10.3390/metabo13030389 (PMC10059836; doi:10.3390/metabo13030389)
Supplement: Supplementary file 1 [file metabolites-13-00389-s001.zip › metabolites-2188193-supplementary.pdf]

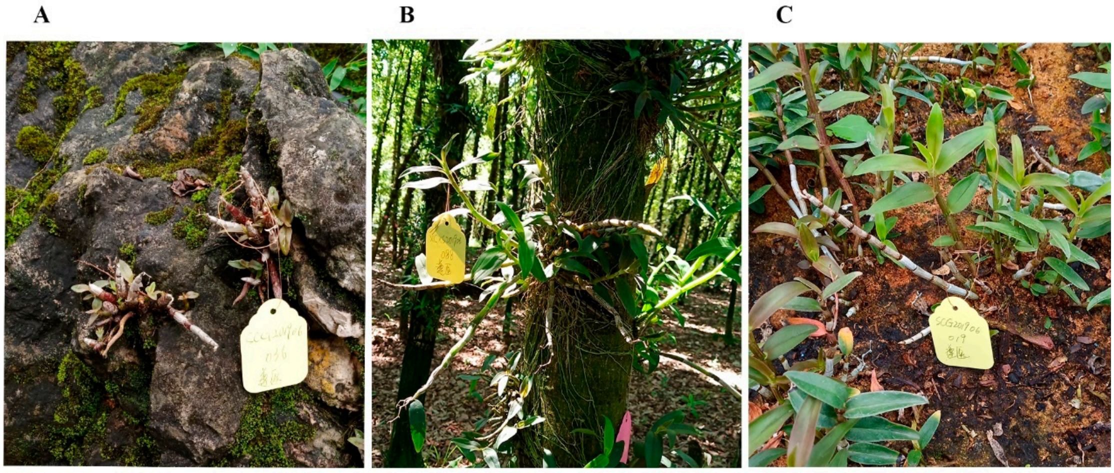

Figure S1: Pictures of Dendrobium samples in different cultivation methods (Note: A is Stone epiphytic cultivation, B is Tree epiphytic cultivation, C is Greenhouse cultivation)

Quality Control Samples (QC) are prepared from a mixture of sample extracts and are used to analyse the reproducibility of the samples under the same processing method. During the instrumental analysis, one QC sample is inserted in every 10 samples analysed for testing to monitor the reproducibility of the analytical process. The graphs below show the Total ions current (TIC, i.e. a continuous plot of the intensity of all ions in the mass spectra at each time point) for the mixed QC samples, the MRM metabolite assay multi-peak plot (XIC for multiple substance extraction), the horizontal coordinate is the Retention time (Rt) for the metabolite assay, and the vertical coordinate is the Retention time (Rt) for the metabolite assay. (Rt), the vertical coordinate is the intensity of the ion flow (intensity in cps, count per second)

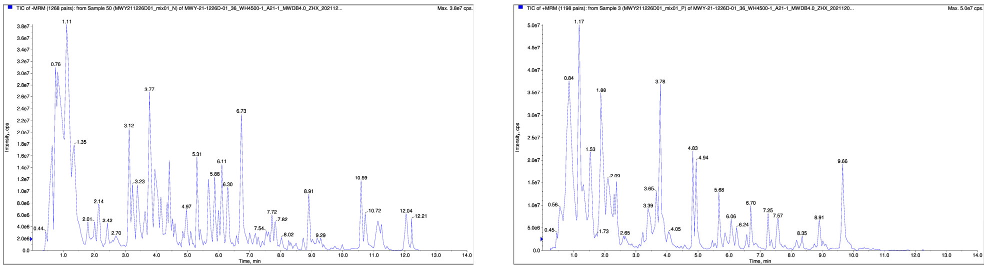

Figure S2: Total ion flow diagram for mass spectrometry of mixed samples (Note: N on the left represents the negative ion mode and P on the right represents the positive ion mode)

Table S1. The 185 differential metabolite information

Re-ID	Molecular Weight (Da)	Formula	Ionization model	Compounds	Class I	Class II	CAS	Comparative Content
17-p	111.043	C4H5N3O	[M+H]+	Isocytosine	Nucleotides and derivatives	Nucleotides and derivatives	108-53-2	Greenhouse
21-p	113.059	C4H7N3O	[M+H]+	Creatinine	Organic acids	Organic acids	60-27-5	Tree
33-p	118.027	C4H6O4	[M-H]-	Methylmalonic acid*	Organic	Organic acids	516-05-2	Stone

					acids			
41-p	120.058	C8H8O	[M-H]-	4-Methylbenzaldehyde*	Others	Others	104-87-0	Tree
56-p	126.032	C6H6O3	[M+H]+	1,3,5-Benzenetriol	Phenolic acids	Phenolic acids	108-73-6	Tree
66-p	130.111	C6H14N2O	[M+H]+	N-Acetylputrescine	Alkaloids	Alkaloids	18233-70-0	Greenhouse
67-p	130.122	C5H14N4	[M+H]+	Agmatine	Alkaloids	Alkaloids	306-60-5	Greenhouse
115-p	144.058	C10H8O	[M-H]-	1-Naphthol	Phenolic acids	Phenolic acids	90-15-3	Tree
144-p	149.048	C8H7NO2	[M+H]+	4-Hydroxymandelonitrile	Alkaloids	Phenolamine	13093-65-7	Tree
146-p	149.07	C6H7N5	[M+H]+	1-Methyladenine	Nucleotides and derivatives	Nucleotides and derivatives	5142-22-3	Tree
153-p	150.068	C9H10O2	[M-H]-	4'-Hydroxypropiophenone	Phenolic acids	Phenolic acids	70-70-2	Tree
155-p	151.049	C5H5N5O	[M+H]+	Isoguanine	Nucleotides and derivatives	Nucleotides and derivatives	3373-53-3	Stone
165-p	152.047	C8H8O3	[M+H]+	2',6'-Dihydroxyacetophenone	Phenolic acids	Phenolic acids	699-83-2	Stone
171-p	152.047	C8H8O3	[M-H]-	2-hydroxymethyl benzoic acid	Phenolic acids	Phenolic acids	612-20-4	Stone
173-p	152.068	C5H12O5	[M-H]-	D-Arabitol*	Others	Saccharides and Alcohols	488-82-4	Tree
178-p	154.027	C7H6O4	[M-H]-	2,3-Dihydroxybenzoic Acid*	Phenolic acids	Phenolic acids	303-38-8	Tree
185-p	158.167	C10H22O	[M-H]-	1-Decanol	Others	Saccharides and Alcohols	112-30-1	Greenhouse
195-p	161.069	C6H11NO4	[M-H]-	N-Acetyl-L-threonine	Amino acids and derivatives	Amino acids and derivatives	17093-74-2	Tree
216-p	166.027	C8H6O4	[M-H]-	Phthalic acid	Phenolic acids	Phenolic acids	88-99-3	Stone
227-p	166.084	C6H14O5	[M-H]-	L-Fucitol	Others	Saccharides and Alcohols	13074-06-1	Greenhouse
233-p	168.042	C8H8O4	[M+H]+	2,6-Dimethoxy-1,4-benzoquinone	Quinones	Quinones	530-55-2	Stone
236-p	168.042	C8H8O4	[M-H]-	Protocatechuic Acid Methyl Ester	Phenolic acids	Phenolic acids	2150-43-8	Tree
237-p	168.09	C8H12N2O2	[M+H]+	Cyclo(L-Ala-L-Pro)	Amino acids and derivatives	Amino acids and derivatives	36357-32-1	Tree
242-p	169.998	C3H7O6P	[M-H]-	DL-Glyceraldehyde-3-	Organic	Organic acids	591-59-3	Tree

				phosphate	acids			
252-p	174.016	C6H6O6	[M-H]-	Dehydroascorbic acid	Others	Vitamin	490-83-5	Tree
263-p	176.032	C6H8O6	[M-H]-	D-Glucurono-6,3-lactone	Others	Saccharides and Alcohols	32449-92-6	Stone
266-p	176.047	C10H8O3	[M-H]-	6-Hydroxy-4-methylcoumarin	Lignans and Coumarins	Coumarins	2373-31-1	Tree
269-p	176.084	C11H12O2	[M+H]+	Ethyl cinnamate	Phenolic acids	Phenolic acids	103-36-6	Stone
275-p	178.063	C10H10O3	[M-H]-	4-Methoxycinnamic acid	Phenolic acids	Phenolic acids	830-09-1	Stone
276-p	178.063	C10H10O3	[M-H]-	3,4-Methylenedioxy cinnamyl alcohol	Lignans and Coumarins	Lignans	58095-76-4	Stone
288-p	180.079	C10H12O3	[M-H]-	Methyl 3-(4-hydroxyphenyl)propionate	Phenolic acids	Phenolic acids	5597-50-2	Greenhouse
290-p	180.115	C11H16O2	[M-H]-	Olivetol	Phenolic acids	Phenolic acids	500-66-3	Tree
291-p	181.074	C9H11NO3	[M+H]+	L-Tyrosine	Amino acids and derivatives	Amino acids and derivatives	60-18-4	Tree
326-p	189.064	C7H11NO5	[M-H]-	N-Acetyl-L-glutamic acid	Amino acids and derivatives	Amino acids and derivatives	1188-37-0	Tree
327-p	189.07	C11H11NO2	[M+H]+	3-Indolepropionic acid	Alkaloids	Plumerane	830-96-6	Tree
330-p	190.095	C7H14N2O4	[M+H]+	2,6-Diaminooimelic acid	Organic acids	Organic acids	583-93-7	Greenhouse
332-p	192.027	C6H8O7	[M-H]-	Citric Acid	Organic acids	Organic acids	77-92-9	Stone
333-p	192.042	C10H8O4	[M+H]+	6,7-Dihydroxy-4-methylcoumarin	Lignans and Coumarins	Coumarins	529-84-0	Stone
334-p	192.042	C10H8O4	[M+H]+	Scopoletin (7-Hydroxy-6-methoxycoumarin)	Lignans and Coumarins	Coumarins	92-61-5	Stone
335-p	192.042	C10H8O4	[M+H]+	Noreugenin; 5,7-Dihydroxy-2-Methylchromone	Phenolic acids	Phenolic acids	1013-69-0	Stone
336-p	192.063	C7H12O6	[M-H]-	Quinic Acid	Organic acids	Organic acids	77-95-2	Stone
337-p	192.079	C11H12O3	[M+H]+	p-Coumaric acid ethyl ester	Phenolic acids	Phenolic acids	7362-39-2	Tree
338-p	193.074	C10H11NO3	[M+H]+	N-(2-Methylbenzoyl)glycine	Amino acids and derivatives	Amino acids and derivatives	42013-20-7	Tree

341-p	194.043	C6H10O7	[M-H]-	D-Glucoronic acid*	Others	Saccharides and Alcohols	6556-12-3	Tree
346-p	194.058	C10H10O4	[M+H]+	Vanillin acetate	Phenolic acids	Phenolic acids	881-68-5	Stone
347-p	194.106	C10H14N2O2	[M+H]+	Cyclo(Pro-Pro)	Amino acids and derivatives	Amino acids and derivatives	6708-06-1	Stone
353-p	196.121	C10H16N2O2	[M+H]+	Cyclo(D-Val-L-Pro)	Amino acids and derivatives	Amino acids and derivatives	27483-18-7	Stone
355-p	198.053	C9H10O5	[M-H]-	Gallic Acid Ethyl Ester; Ethyl gallate	Phenolic acids	Phenolic acids	831-61-8	Tree
356-p	198.053	C9H10O5	[M-H]-	Syringic acid	Phenolic acids	Phenolic acids	530-57-4	Tree
359-p	202.111	C12H14N2O	[M+H]+	Acetryptine	Alkaloids	Plumerane	3551-18-6	Stone
360-p	202.121	C10H18O4	[M-H]-	Sebacate	Organic acids	Organic acids	111-20-6	Stone
361-p	202.136	C14H18O	[M+H]+	2-Pentyl-3-phenyl-2-propenal	Others	Others	122-40-7	Stone
362-p	202.143	C8H18N4O2	[M+H]+	N,N'-Dimethylarginine;SDMA	Amino acids and derivatives	Amino acids and derivatives	30344-00-4	Stone
363-p	202.143	C8H18N4O2	[M+H]+	NG,NG-Dimethyl-L-arginine	Amino acids and derivatives	Amino acids and derivatives	30315-93-6	Tree
364-p	202.216	C10H26N4	[M+H]+	Spermine	Alkaloids	Alkaloids	71-44-3	Tree
369-p	206.131	C13H18O2	[M+H]+	4-Hydroxy-3,5-diisopropylbenzaldehyde	Phenolic acids	Phenolic acids	10537-86-7	Tree
370-p	206.167	C14H22O	[M-H]-	2,6-Di-tert-butylphenol*	Phenolic acids	Phenolic acids	128-39-2	Tree
371-p	206.167	C14H22O	[M-H]-	2,4-Di-Tert-Butylphenol*	Phenolic acids	Phenolic acids	96-76-4	Stone
372-p	207.09	C11H13NO3	[M-H]-	N-Acetyl-L-phenylalanine	Amino acids and derivatives	Amino acids and derivatives	2018-61-3	Tree
373-p	208.037	C10H8O5	[M+H]+	Fraxetin (7,8-Dihydroxy-6-methoxycoumarin)	Lignans and Coumarins	Coumarins	574-84-5	Tree
375-p	208.074	C11H12O4	[M+H]+	4-Acetoxy-3-Ethoxybenzaldehyde	Others	Others	72207-94-4	Tree
376-p	208.074	C11H12O4	[M-H]-	Sinapinaldehyde	Phenolic acids	Phenolic acids	4206-58-0	Tree
377-p	208.074	C11H12O4	[M+H]+	3,4-Dimethoxycinnamic acid	Phenolic	Phenolic acids	2316-26-	Tree

					acids		9	
378-p	208.074	C11H12O4	[M-H]-	Ferulic acid methyl ester	Phenolic acids	Phenolic acids	2309-07-1	Tree
379-p	210.038	C6H10O8	[M-H]-	D-Galactaric acid*	Others	Saccharides and Alcohols	526-99-8	Tree
384-p	210.089	C11H14O4	[M-H]-	Sinapyl alcohol*	Phenolic acids	Phenolic acids	537-33-7	Stone
386-p	210.137	C11H18N2O2	[M+H]+	Cyclo(Pro-Leu)	Amino acids and derivatives	Amino acids and derivatives	5654-86-4	Tree
387-p	210.161	C13H22O2	[M+H]+	Blumenol C	Terpenoids	Terpene	36151-02-7	Tree
392-p	214.024	C5H11O7P	[M-H]-	2-Deoxyribose-5'-phosphate	Nucleotides and derivatives	Nucleotides and derivatives	102916-66-5	Tree
393-p	214.099	C14H14O2	[M-H]-	Dihydropinosylvin	Phenolic acids	Phenolic acids	14531-52-3	Tree
398-p	219.111	C9H17NO5	[M-H]-	D-Pantothenic Acid	Others	Vitamin	79-83-4	Tree
399-p	221.09	C8H15NO6	[M+H]+	N-Acetyl-D-glucosamine	Others	Saccharides and Alcohols	7512-17-6	Tree
401-p	222.053	C11H10O5	[M+H]+	Isofraxidin	Lignans and Coumarins	Coumarins	486-21-5	Tree
404-p	222.1	C11H14N2O3	[M+H]+	L-Glycyl-L-phenylalanine	Amino acids and derivatives	Amino acids and derivatives	721-66-4	Tree
405-p	222.198	C15H26O	[M+H]+	Elemol	Terpenoids	Sesquiterpenoids	639-99-6	Tree
407-p	224.032	C10H8O6	[M-H]-	Sideretin (5,7,8-Trihydroxy-6-methoxycoumarin)	Lignans and Coumarins	Coumarins	-	Tree
410-p	225.101	C12H11N5	[M-H]-	6-Benzylaminopurine	Nucleotides and derivatives	Nucleotides and derivatives	1214-39-7	Stone
415-p	226.132	C11H18N2O3	[M+H]+	Cyclo(L-Leu-trans-4-hydroxy-L-Pro)	Amino acids and derivatives	Amino acids and derivatives	115006-86-5	Greenhouse
420-p	228.079	C14H12O3	[M-H]-	Resveratrol	Others	Stilbene	501-36-0	Tree
421-p	228.115	C15H16O2	[M+H]+	Dihydropinosylvin methyl ether	Phenolic acids	Phenolic acids	17635-59-5	Stone
422-p	228.209	C14H28O2	[M-H]-	Myristic Acid	Lipids	Free fatty acids	544-63-8	Stone
429-p	236.116	C12H16N2O3	[M+H]+	L-Alanyl-L-Phenylalanine	Amino acids and derivatives	Amino acids and derivatives	3061-90-3	Stone
432-p	238.084	C12H14O5	[M-H]-	1-O-p-Cumaroylglycerol	Phenolic	Phenolic acids	106055-	Stone

					acids		11-2	
433-p	240.079	C15H12O3	[M-H]-	Chrysophanol-9-anthrone	Quinones	Anthraquinone	491-58-7	Stone
435-p	240.245	C16H32O	[M-H]-	Palmitaldehyde	Lipids	Free fatty acids	629-80-1	Stone
437-p	242.069	C13H10N2O3	[M+H]+	1-Methoxycarbonyl-β-Carboline-N-Oxide	Alkaloids	Plumerane	74690-74-7	Stone
438-p	242.08	C12H10N4O2	[M+H]+	Lumichrome	Alkaloids	Alkaloids	1086-80-2	Stone
439-p	242.09	C10H14N2O5	[M+H]+	Thymidine	Nucleotides and derivatives	Nucleotides and derivatives	50-89-5	Stone
440-p	242.093	C15H14O3	[M+H]+	Hircinol	Quinones	PhenAnthraquinones	41060-05-3	Stone
442-p	242.094	C15H14O3	[M+H]+	Coelonin	Quinones	PhenAnthraquinones	82344-82-9	Stone
443-p	242.094	C15H14O3	[M+H]+	Lusianthridin	Quinones	PhenAnthraquinones	87530-30-1	Stone
445-p	242.225	C15H30O2	[M-H]-	Pentadecanoic Acid	Lipids	Free fatty acids	1002-84-2	Stone
447-p	243.086	C9H13N3O5	[M+H]+	Cytarabine	Nucleotides and derivatives	Nucleotides and derivatives	147-94-4	Stone
449-p	244.07	C9H12N2O6	[M-H]-	β-Pseudouridine	Nucleotides and derivatives	Nucleotides and derivatives	1445-07-4	Stone
451-p	244.07	C9H12N2O6	[M-H]-	Uridine	Nucleotides and derivatives	Nucleotides and derivatives	58-96-8	Tree
452-p	244.121	C14H16N2O2	[M+H]+	Cyclo(Pro-Phe)	Amino acids and derivatives	Amino acids and derivatives	3705-26-8	Tree
454-p	244.167	C13H24O4	[M-H]-	Tridecanedioic acid	Lipids	Free fatty acids	505-52-2	Stone
455-p	244.179	C12H24N2O3	[M+H]+	L-Leucyl-L-Leucine	Amino acids and derivatives	Amino acids and derivatives	3303-31-9	Stone
456-p	246.1	C13H14N2O3	[M+H]+	N-(3-Indolylacetyl)-L-alanine	Amino acids and derivatives	Amino acids and derivatives	57105-39-2	Stone
5-n	252.147	C13H20N2O3	[M+H]+	Dihydrocaffeoylputrescine	Alkaloids	Phenolamine	-	Stone
9-n	256.037	C14H8O5	[M-H]-	1,2,4-Trihydroxyanthraquinone	Quinones	Anthraquinone	81-54-9	Stone
17-n	258.085	C10H14N2O6	[M-H]-	5-Methyluridine	Nucleotides and derivatives	Nucleotides and derivatives	1463-10-1	Stone
18-n	258.183	C14H26O4	[M-H]-	1,14-Tetradecanedioic Acid	Lipids	Free fatty acids	821-38-5	Stone

27-n	265.112	C12H17N4OS+	[M]+	Thiamine (Vitamin B1)	Others	Vitamin	70-16-6	Tree
28-n	266.069	C15H10N2O3	[M+H]+	3-(2'-Hydroxyl-phenyl)-4-(3H)-quinazolone	Alkaloids	Alkaloids	-	Stone
58-n	274.121	C16H18O4	[M+H]+	Dendrobin A	Alkaloids	Alkaloids	-	Tree
60-n	276.209	C18H28O2	[M+H]+	Stearidonic acid	Lipids	Free fatty acids	20290-75-9	Tree
61-n	278.152	C16H22O4	[M+H]+	Butyl isobutyl phthalate*	Phenolic acids	Phenolic acids	17851-53-5	Tree
64-n	278.163	C15H22N2O3	[M+H]+	L-Leucyl-L-phenylalanine	Amino acids and derivatives	Amino acids and derivatives	56217-82-4	Tree
65-n	278.193	C17H28NO2+	[M+H]+	N-Methyldendrobine	Alkaloids	Sesquiterpene alkaloids	-	Stone
80-n	281.112	C11H15N5O4	[M+H]+	N6-methyladenosine	Nucleotides and derivatives	Nucleotides and derivatives	1867-73-8	Tree
86-n	283.092	C10H13N5O5	[M+H]+	Guanosine	Nucleotides and derivatives	Nucleotides and derivatives	118-00-3	Tree
89-n	283.121	C17H17NO3	[M+H]+	p-Coumaroyltyramine	Alkaloids	Phenolamine	36417-86-4	Greenhouse
95-n	284.105	C17H16O4	[M+H]+	5,7-Dimethoxyflavone	Flavonoids	Flavones	1036-72-2	Greenhouse
98-n	286.048	C15H10O6	[M+H]+	Kaempferol (3,5,7,4'-Tetrahydroxyflavone)	Flavonoids	Flavonols	520-18-3	Tree
99-n	286.048	C15H10O6	[M+H]+	6-Demethoxycapillarisin	Others	Others	61854-36-2	Tree
100-n	286.084	C16H14O5	[M+H]+	Isooxypeucedanine	Lignans and Coumarins	Coumarins	5058-15-1	Tree
102-n	286.214	C16H30O4	[M-H]-	Hexadecanedioic acid	Lipids	Free fatty acids	505-54-4	Stone
103-n	286.23	C20H30O	[M-H]-	Ferruginol	Terpenoids	Ditepenoids	514-62-5	Stone
104-n	286.251	C17H34O3	[M-H]-	3-Hydroxy-palmitic acid methyl ester	Lipids	Free fatty acids	51883-36-4	Stone
113-n	290.04	C7H15O10P	[M-H]-	D-Sedoheptuiose 7-phosphate	Others	Saccharides and Alcohols	2646-35-7	Tree
114-n	290.115	C16H18O5	[M-H]-	Dendrocandin C	Phenolic acids	Phenolic acids	-	Tree
134-n	298.324	C20H42O	[M-H]-	1-Eicosanol	Lipids	Free fatty acids	629-96-9	Tree
137-n	300.063	C16H12O6	[M+H]+	Rhamnocitrin (7-Methylkaempferol)*	Flavonoids	Flavonols	569-92-6	Stone
143-n	300.1	C17H16O5	[M-H]-	4'-Hydroxy-5,7-dimethoxyflavanone	Flavonoids	Flavanones	-	Tree
144-n	300.121	C14H20O7	[M-H]-	Salidroside	Phenolic	Phenolic acids	10338-	Tree

					acids		51-9	
145-n	300.135	C18H20O4	[M-H]-	Doitungbiphenyl A	Phenolic acids	Phenolic acids	-	Stone
161-n	304.058	C15H12O7	[M-H]-	Taxifolin(Dihydroquercetin)	Flavonoids	Flavanonols	480-18-2	Stone
163-n	304.131	C17H20O5	[M+H]+	Moscatilin (4,4'-Dihydroxy-3,3',5-Trimethoxybibenzyl)	Phenolic acids	Phenolic acids	108853-14-1	Stone
174-n	312.147	C18H20N2O3	[M+H]+	L-Phenylalanyl-L-phenylalanine	Amino acids and derivatives	Amino acids and derivatives	2577-40-4	Stone
183-n	313.131	C18H19NO4	[M+H]+	N-Cis-Feruloyltyramine	Alkaloids	Phenolamine	80510-09-4	Stone
187-n	314.246	C18H34O4	[M-H]-	12,13-DHOME; (9Z)-12,13-Dihydroxyoctadec-9-enoic acid	Lipids	Free fatty acids	263399-35-5	Tree
216-n	330.241	C18H34O5	[M-H]-	9,12,13-TriHOME; 9(S),12(S),13(S)-Trihydroxy-10(E)-octadecenoic acid	Lipids	Free fatty acids	97134-11-7	Stone
241-n	343.142	C19H21NO5	[M+H]+	N-Trans-Feruloyl-3'-O-methyldopamine	Alkaloids	Phenolamine	-	Tree
286-n	416.111	C21H20O9	[M+H]+	Apigenin-6-C-fucoside	Flavonoids	Flavonoid carbonoside	-	Stone
288-n	418.148	C18H26O11	[M-H]-	2-Hydroxyphenol-1-O-glucosyl(6→1)rhamnoside	Phenolic acids	Phenolic acids	-	Stone
291-n	422.083	C12H23O14P	[M-H]-	Sucrose-6-phosphate	Others	Saccharides and Alcohols	-	Stone
297-n	432.106	C21H20O10	[M-H]-	Apigenin-8-C-Glucoside (Vitexin)*	Flavonoids	Flavonoid carbonoside	3681-93-4	Stone
308-n	442.09	C22H18O10	[M-H]-	Epicatechin gallate*	Flavonoids	Flavanols	1257-08-5	Stone
311-n	444.303	C31H40O2	[M+H]+	Menatetrenone (Vitamin K2)	Others	Vitamin	11032-49-8	Stone
312-n	446.215	C21H34O10	[M-H]-	Dendroside G	Terpenoids	Sesquiterpenoids	503443-66-1	Stone
313-n	448.101	C21H20O11	[M+H]+	Kaempferol-3-O-glucoside (Astragalin)*	Flavonoids	Flavonols	480-10-4	Stone
314-n	448.101	C21H20O11	[M+H]+	Luteolin-3'-O-glucoside	Flavonoids	Flavones	5154-41-6	Stone
338-n	464.132	C22H24O11	[M-H]-	Hesperetin-5-O-glucoside	Flavonoids	Flavanones	69651-80-5	Stone
345-n	472.191	C22H34NO10+	[M]+	Sinapine-4-O-Glucoside	Alkaloids	Phenolamine	-	Stone
346-n	472.355	C30H48O4	[M-H]-	Maslinic acid	Terpenoids	Triterpene	4373-41-5	Stone
350-n	472.355	C30H48O4	[M-H]-	2α-Hydroxyursolic acid	Terpenoids	Triterpene	-	Stone
351-n	473.166	C20H23N7O7	[M+H]+	10-Formyltetrahydrofolic	Alkaloids	Alkaloids	2800-34-	Stone

				Acid			2	
352-n	475.184	C24H29NO9	[M-H]-	N-(4-O-(Glucosyl)-E-feruloyl)-tyramine	Alkaloids	Phenolamine	-	Tree
370-n	491.301	C24H46NO7P	[M+H]+	LysoPC 16:2	Lipids	LPC	-	Stone
371-n	491.301	C24H46NO7P	[M+H]+	LysoPC 16:2(2n isomer)	Lipids	LPC	-	Stone
372-n	492.127	C23H24O12	[M+H]+	Tricin-7-O-Glucoside	Flavonoids	Flavones	32769-01-0	Greenhouse
373-n	493.317	C24H48NO7P	[M+H]+	LysoPC 16:1	Lipids	LPC	76790-27-7	Stone
375-n	495.332	C24H50NO7P	[M+H]+	LysoPC 16:0	Lipids	LPC	17364-16-8	Stone
376-n	495.332	C24H50NO7P	[M+H]+	LysoPC 16:0(2n isomer)	Lipids	LPC	-	Greenhouse
378-n	501.286	C25H44NO7P	[M+H]+	LysoPE 20:4(2n isomer)	Lipids	LPE	-	Tree
380-n	503.301	C25H46NO7P	[M+H]+	LysoPE 20:3	Lipids	LPE	-	Greenhouse
381-n	504.127	C24H24O12	[M-H]-	1,6-Di-O-caffeoyl-β-D-glucose	Phenolic acids	Phenolic acids	-	Stone
388-n	504.345	C30H48O6	[M-H]-	Madecassic acid	Terpenoids	Triterpene	18449-41-7	Greenhouse
394-n	508.179	C21H32O14	[M-H]-	6'-O-Glucosylaucubin	Others	Others	-	Tree
396-n	509.348	C25H52NO7P	[M+H]+	LysoPC 17:0	Lipids	LPC	50930-23-9	Stone
397-n	514.314	C27H46O9	[M+H]+	1-α-Linolenoyl-glycerol-3-O-glucoside	Lipids	Glycerol ester	-	Stone
406-n	520.194	C26H32O11	[M+H]+	Dehydrodiconiferyl alcohol-gamma'-O-glucoside	Phenolic acids	Phenolic acids	-	Tree
407-n	520.194	C26H32O11	[M+H]+	Dehydrodiconiferyl alcohol-4-O-glucoside	Others	Others	107870-88-2	Stone
416-n	524.226	C26H36O11	[M-H]-	Secoisolariciresinol 4-O-glucoside	Lignans and Coumarins	Lignans	-	Stone
417-n	530.194	C31H30O8	[M-H]-	Dendrocandin J	Others	Others	1820612-27-8	Stone
422-n	543.332	C28H50NO7P	[M+H]+	LysoPC 20:4	Lipids	LPC	-	Greenhouse
431-n	550.204	C27H34O12	[M-H]-	5'-Methoxymatairesinoside	Lignans and Coumarins	Lignans	1691201-82-7	Stone
433-n	564.123	C26H28O14	[M+H]+	Isoschaftoside	Flavonoids	Flavonoid carbonoside	52012-29-0	Stone
434-n	564.123	C26H28O14	[M+H]+	Schaftoside	Flavonoids	Flavonoid carbonoside	51938-32-0	Stone
441-n	567.332	C30H50NO7P	[M+H]+	LysoPC 22:6	Lipids	LPC	-	Stone
442-n	569.348	C30H52NO7P	[M+H]+	LysoPC 22:5(2n isomer)	Lipids	LPC	-	Stone
446-n	578.164	C27H30O14	[M+H]+	Isovitexin-2''-O-rhamnoside	Flavonoids	Flavonoid carbonoside	-	Stone

447-n	578.164	C27H30O14	[M+H]+	Violanthin	Flavonoids	Flavonoid carbonoside	40581-17-7	Stone
448-n	580.179	C27H32O14	[M-H]-	Naringenin-7-O-Neohesperidoside(Naringin)*	Flavonoids	Flavanones	10236-47-2	Stone
450-n	580.216	C28H36O13	[M-H]-	Syringaresinol-4'-O-glucoside	Lignans and Coumarins	Lignans	7374-79-0	Tree
460-n	594.158	C27H30O15	[M+H]+	Apigenin-6,8-di-C-glucoside (Vicenin-2)	Flavonoids	Flavonoid carbonoside	23666-13-9	Stone
466-n	610.153	C27H30O16	[M+H]+	Quercetin-3-O-rutinoside (Rutin)	Flavonoids	Flavonols	153-18-4	Stone
473-n	624.169	C28H32O16	[M+H]+	Chrysoeriol-6,8-di-C-glucoside	Flavonoids	Flavonoid carbonoside	-	Tree
474-n	624.169	C28H32O16	[M+H]+	Hispidulin-8-C-(2''-O-glucosyl)glucoside	Flavonoids	Flavonoid carbonoside	-	Stone
478-n	650.142	C29H30O17	[M+H]+	Apigenin-7-O-(2''-O-apiosyl)(6''-Malonyl)glucoside	Flavonoids	Flavones	-	Stone
479-n	652.164	C29H32O17	[M-H]-	Kaempferol-3-O-(6''-Acetyl)glucosyl-(1→3)-Galactoside	Flavonoids	Flavonols	-	Stone
480-n	666.222	C24H42O21	[M-H]-	Stachyose	Others	Saccharides and Alcohols	470-55-3	Stone
482-n	666.222	C24H42O21	[M-H]-	Nystose	Others	Saccharides and Alcohols	13133-07-8	Stone
488-n	700.258	C32H44O17	[M-H]-	Olivil-4,4'-Di-O-glucoside	Lignans and Coumarins	Lignans	-	Stone

Re-ID is a ranking of compounds based on their molecular weight and the results of positive and negative ion scanning patterns

Table S2. Compounds reported in the literature to be contained in *D. officinale*

No.	Description from best library fit	Classification	
1	Oleate (3TMS)	Fatty acids	[1]
2	Octadecanoate (2TMS)	Fatty acids	[1]
3	n-Pentadecanoate (1 TMS)	Fatty acids	[1]
4	Tetradecanoate (1TMS)	Fatty acids	[1]
5	Hexadecanoate (1TMS)	Fatty acids	[1]
6	2-Monopalmitoylglycerol (1TMS)	Fatty acids	[1]
7	Trehalose (8TMS)	Sugars and glycosides	[1]
8	Mannobiose (8TMS)	Sugars and glycosides	[1]
9	Sucrose (8TMS)	Sugars and glycosides	[1]

10	Cellobiose (8TMS)	Sugars and glycosides	[1]
11	Glucose (5TMS)	Sugars and glycosides	[1]
12	Galactose (5TMS)	Sugars and glycosides	[1]
13	Mannose (5TMS)	Sugars and glycosides	[1]
14	Ribose (4TMS)	Sugars and glycosides	[1]
15	Lyxofuranose (4TMS)	Sugars and glycosides	[1]
16	Fructose (4TMS)	Sugars and glycosides	[1]
17	Sorbopyranose (5TMS)	Sugars and glycosides	[1]
18	Levoglucosan (3TMS)	Sugars and glycosides	[1]
19	Xylose (4TMS)	Sugars and glycosides	[1]
20	Erythrose (3TMS)	Sugars and glycosides	[1]
21	Arabinose (4TMS)	Sugars and glycosides	[1]
22	Sedoheptulose (6TMS)	Sugars and glycosides	[1]
23	Allose (5TMS)	Sugars and glycosides	[1]
24	Maltose (8TMS)	Sugars and glycosides	[1]
25	Tagatose (5TMS)	Sugars and glycosides	[1]
26	Turanose	Sugars and glycosides	[1]
27	Talose (5TMS)	Sugars and glycosides	[1]
28	Rhamnopyranose (4TMS)	Sugars and glycosides	[1]
29	Glucopyranoside (4TMS)	Sugars and glycosides	[1]
30	2-0-Glycerol-a-d-galactopyranoside (6TMS)	Sugars and glycosides	[1]
31	Mannopyranoside (4TMS)	Sugars and glycosides	[1]
32	Sorbopyranoside (4TMS)	Sugars and glycosides	[1]
33	2-Keto-gluconate (5TMS)	Organic acids	[1]
34	Gluconate (5TMS)	Organic acids	[1]
35	Glucuronate (4TMS)	Organic acids	[1]
36	Glucarate (4TMS)	Organic acids	[1]
37	Galactarate (4TMS)	Organic acids	[1]
38	Pyruvate (2TMS)	Organic acids	[1]
39	Fumarate	Organic acids	[1]
40	2-ketoglutarate (3TMS)	Organic acids	[1]
41	Threonate (3TMS)	Organic acids	[1]
42	Mannonate (4TMS)	Organic acids	[1]
43	Ribonate (4TMS)	Organic acids	[1]
44	Benzoate	Organic acids	[1]
45	Phenylacetate	Organic acids	[1]
46	Succinate (2TMS)	Organic acids	[1]
47	Tartarate (2TMS)	Organic acids	[1]
48	Oxalate	Organic acids	[1]
49	Butanoate	Organic acids	[1]
50	Propanoate	Organic acids	[1]
51	Pentanoate	Organic acids	[1]
52	Hexanoate	Organic acids	[1]

53	Acetate	Organic acids	[1]
54	Nonanoate	Organic acids	[1]
55	Carbamate	Organic acids	[1]
56	Phthalate	Organic acids	[1]
57	lsophthalate	Organic acids	[1]
58	Terephthalate	Organic acids	[1]
59	1-Naphthalenesulfonate	Organic acids	[1]
60	2-Butenedioate (2TMS)	Organic acids	[1]
61	2-Butenoate	Organic acids	[1]
62	Glutarate	Organic acids	[1]
63	1-Cyclohexene-1 -carboxylic acid (3TMS)	Organic acids	[1]
64	trans-2,3-Dimethylacrylic acid	Organic acids	[1]
65	Citrate (3TMS)	Organic acids	[1]
66	Galacturonate (5TMS)	Organic acids	[1]
67	Arabino-Hexonate (4TMS)	Organic acids	[1]
68	Alanine	Amino acids	[1]
69	Valine	Amino acids	[1]
70	Norleucine	Amino acids	[1]
71	Isoleucine (1 TMS)	Amino acids	[1]
72	L eucine	Amino acids	[1]
73	Proline (1TMS)	Amino acids	[1]
74	Glycine (2TMS)	Amino acids	[1]
75	Serine (2TMS)	Amino acids	[1]
76	Threonine (3TMS)	Amino acids	[1]
77	Aspartate (2TMS)	Amino acids	[1]
78	Glutamate (2TMS)	Amino acids	[1]
79	Glutamine (3TMS)	Amino acids	[1]
80	Asparagine	Amino acids	[1]
81	Phenylalanine (2TMS)	Amino acids	[1]
82	Methionine	Amino acids	[1]
83	Allylglycine	Amino acids	[1]
84	Ethanamine	Amines and amides	[1]
85	Triethylamine	Amines and amides	[1]
86	1,4-Butanediamine (4TMS)	Amines and amides	[1]
87	Benzamide	Amines and amides	[1]
88	Benzenesulfonamide	Amines and amides	[1]
89	Lysergamide	Amines and amides	[1]
90	Hexadecanamide	Amines and amides	[1]
91	Octadecanamide	Amines and amides	[1]
92	Glycerol (3TMS)	Alcohols	[1]
93	Threitol (4TMS)	Alcohols	[1]
94	Galactitol (6TMS)	Alcohols	[1]
95	Arabitol	Alcohols	[1]

96	Myo-inositol (6TMS)	Alcohols	[1]
97	Glucitol	Alcohols	[1]
98	Mannitol (6TMS)	Alcohols	[1]
99	1,2-Propanediol	Alcohols	[1]
100	1,2-Butanediol (2TMS)	Alcohols	[1]
101	Benzenemethanol	Alcohols	[1]
102	Ethanol	Alcohols	[1]
103	Ethane	Alkanes	[1]
104	Butane	Alkanes	[1]
105	Hexane	Alkanes	[1]
106	Heptane	Alkanes	[1]
107	Octane	Alkanes	[1]
108	Decane	Alkanes	[1]
109	Nonane	Alkanes	[1]
110	Undecane	Alkanes	[1]
111	Dodecane	Alkanes	[1]
112	Tridecane	Alkanes	[1]
113	Tetradecane	Alkanes	[1]
114	Pentadecane	Alkanes	[1]
115	Hexadecane	Alkanes	[1]
116	Heptadecane	Alkanes	[1]
117	Nonadecane	Alkanes	[1]
118	Eicosane	Alkanes	[1]
119	Heneicosane	Alkanes	[1]
120	Tetracosane	Alkanes	[1]
121	Heptacosane	Alkanes	[1]
122	Ethanone	Ketones	[1]
123	Psicose (6TMS)	Ketones	[1]
124	Propan-1-one	Ketones	[1]
125	Sulfurous acid	Others	[1]
126	Phosphate (3:1)	Others	[1]
127	Benzene	Others	[1]
128	Urea	Others	[1]
129	Phenol	Others	[1]
130	Piperidine	Others	[1]
131	a-Pinene	Others	[1]
132	Cadaverine (3TMS)	Others	[1]
133	Oleanitrile	Others	[1]
134	1-Piperidinecarboxaldehyde	Others	[1]
135	Benzonitrile	Others	[1]
136	Coumarin	Others	[1]
137	1,2-Benzenediol	Others	[1]
138	Quinoline	Others	[1]
139	Thiophene	Others	[1]

140	Apigenin-6,8-di-C-β-d-glucoside	flavonoid	[2]
141	Apigenin-6-C- β -d-xyloside-8-C- β -d-glucoside	flavonoid	[2]
142	Isoschaftoside	flavonoid	[2]
143	Schaftoside	flavonoid	[2]
144	Apigenin-6-C- β -d-glucoside-8-C- β -d-xyloside	flavonoid	[2]
145	Rutin	flavonoid	[2]
146	Isoviolanthin	flavonoid	[2]
147	Violanthin	flavonoid	[2]
148	Naringin	flavonoid	[2]
149	2-O-acetylglucomannan	Monosaccharide compositions	[3]
150	DP	Monosaccharide compositions	[3]
151	DOP-1	Monosaccharide compositions	[3]
152	DOP-2	Monosaccharide compositions	[3]
153	2,3-O-acetyl-glucomannan	Monosaccharide compositions	[3]
154	DOP-W3-b	Monosaccharide compositions	[3]
155	DOP-I-1	Monosaccharide compositions	[3]
156	DOPa	Monosaccharide compositions	[3]
157	DOPb	Monosaccharide compositions	[3]
158	DOP-1-1	Monosaccharide compositions	[3]
159	DOPA-1	Monosaccharide compositions	[3]
160	DOPA-2	Monosaccharide compositions	[3]
161	S32	Monosaccharide compositions	[3]
162	DWDOP1	Monosaccharide compositions	[3]
163	DWDOP2	Monosaccharide compositions	[3]
164	DWDOP3	Monosaccharide compositions	[3]
165	FWDOP1	Monosaccharide compositions	[3]
166	FWDOP2	Monosaccharide compositions	[3]
167	FWDOP3	Monosaccharide compositions	[3]
168	DOP-40	Monosaccharide compositions	[3]
169	DOP-50	Monosaccharide compositions	[3]
170	DOP-60	Monosaccharide compositions	[3]
171	DOP-70	Monosaccharide compositions	[3]
172	DOPA-1	Monosaccharide compositions	[3]
173	DO	Monosaccharide compositions	[3]
174	DOP1-DES	Monosaccharide compositions	[3]
175	DOP2-DES	Monosaccharide compositions	[3]
176	DOPHWE	Monosaccharide compositions	[3]
177	DOPFTCP	Monosaccharide compositions	[3]
178	DOPCP	Monosaccharide compositions	[3]
179	DOPUHWE	Monosaccharide compositions	[3]
180	DOPMHWE	Monosaccharide compositions	[3]
181	DOPEHWE	Monosaccharide compositions	[3]

182	LDOP-1	Monosaccharide compositions	[3]
183	DOPW-1	Monosaccharide compositions	[3]
184	DOPW-2	Monosaccharide compositions	[3]
185	4-hydroxybenzoic acid	phenolic	[4]
186	vanillic acid	phenolic	[4]
187	syringic acid	phenolic	[4]
188	ferulic acid	phenolic	[4]
189	l-Valine	Alkaloids	[5]
190	l-Isoleucine	Alkaloids	[5]
191	4-Hydroxy-l-threonine	Alkaloids	[5]
192	Urocanic acid	Alkaloids	[5]
193	Tropine or its isomers	Alkaloids	[5]
194	2,3-Dihydroxynortropane or its isomers	Alkaloids	[5]
195	4-Guanidinobutanoic acid	Alkaloids	[5]
196	l-Lysine	Alkaloids	[5]
197	2-Hydroxyadenine	Alkaloids	[5]
198	Cyclocimipronidine	Alkaloids	[5]
199	l-Histidine	Alkaloids	[5]
200	N-tert-Butyloxycarbonyl guanidine	Alkaloids	[5]
201	l-Phenylalanine	Alkaloids	[5]
202	l-Arginine	Alkaloids	[5]
203	β-Phenyl-γ-aminobutyric acid	Alkaloids	[5]
204	l-Homophenylalanine	Alkaloids	[5]
205	l-Tyrosine	Alkaloids	[5]
206	Ng-Methyl-l-arginine	Alkaloids	[5]
207	Alanyl-isoleucine or its isomers	Alkaloids	[5]
208	l-Tryptophan	Alkaloids	[5]
209	Nα -Acetyl-l-arginine	Alkaloids	[5]
210	Valyl-valine	Alkaloids	[5]
211	Anapheline	Alkaloids	[5]
212	2-Amino-3-tetradecanol	Alkaloids	[5]
213	Valyl-leucine or its isomers	Alkaloids	[5]
214	Nδ -Benzoylornithine	Alkaloids	[5]
215	Carbetamide	Alkaloids	[5]
216	Cytidine	Alkaloids	[5]
217	Leucyl-isoleucine or its isomers	Alkaloids	[5]
218	Prolyl-histidine	Alkaloids	[5]
219	Phenylalanyl-valine or its isomers	Alkaloids	[5]
220	Adenosine	Alkaloids	[5]
221	2-Amino-1,3-hexadecanediol	Alkaloids	[5]
222	Isoleucyl-phenylalanine or its isomers	Alkaloids	[5]
223	1-Methyladenosine	Alkaloids	[5]
224	Guanosine	Alkaloids	[5]
225	2-Amino-1,3,4-hexadecanetriol	Alkaloids	[5]

226	N2-(3-Hydroxysuccinoyl)arginine	Alkaloids	[5]
227	Isoleucyl-tyrosine or its isomers	Alkaloids	[5]
228	Alamaridine	Alkaloids	[5]
229	4-O-β-d-Glucopyranoside-	Alkaloids	[5]
230	2,4-benzoxazolediol	Alkaloids	[5]
231	2-Amino-1,3,4-octadecanetriol	Alkaloids	[5]
232	5′-O-beta-d-Glucosylpyridoxine	Alkaloids	[5]
233	N2-Fructopyranosylarginine	Alkaloids	[5]
234	1,2-Dihydro-O-methyltazettine	Alkaloids	[5]
235	Passicapsin	Alkaloids	[5]
236	Meefarnine B	Alkaloids	[5]
237	O-(tri-O-Acetyl- α -l-rhamnopyranoside)-(4-hydroxybenzyl) methylcarbamic acid	Alkaloids	[5]
238	Methyllagerine N-oxide	Alkaloids	[5]

1. Q, J.; C, J.; S, S.; C, S.; Y, C.; Y, L.; H, F.; Y, Z. Metabolic Analysis of Medicinal Dendrobium officinale and Dendrobium huoshanense during Different Growth Years. *PloS one* **2016**, *11*, e0146607, doi:10.1371/journal.pone.0146607.
2. Zhou, C.; Xie, Z.; Lei, Z.; Huang, Y.; Wei, G. Simultaneous identification and determination of flavonoids in Dendrobium officinale. *Chemistry Central journal* **2018**, *12*, 40, doi:10.1186/s13065-018-0403-8.
3. Chen，WH; Wu，JJ; Li，XF; Lu，JM; Wu，W; Sun，YQ; Zhu，B; Qin，LP. Isolation, structural properties, bioactivities of polysaccharides from Dendrobium officinale Kimura et. Migo: A review. *International journal of biological macromolecules* **2021**, *184*, 1000-1013, doi:10.1016/j.ijbiomac.2021.06.156.
4. Yoo, S.R.; Jeong, S.J.; Lee, N.R.; Shin, H.K.; Seo, C.S. Simultaneous determination and anti-inflammatory effects of four phenolic compounds in Dendrobii Herba. *Natural product research* **2017**, *31*, 2923-2926, doi:10.1080/14786419.2017.1300798.
5. Song, C.; Zhang, Y.; Manzoor, M.A.; Li, G. Identification of alkaloids and related intermediates of Dendrobium officinale by solid-phase extraction coupled with high-performance liquid chromatography tandem mass spectrometry. *Frontiers in plant science* **2022**, *13*, 952051, doi:10.3389/fpls.2022.952051.
